# Phylogeny, Synteny, and Distribution of Type III Secretion Systems in *Burkholderia cenocepacia*: A Closer Look Into Host Span

**DOI:** 10.1002/mbo3.70101

**Published:** 2025-11-02

**Authors:** Gabrielle Tomé Cordeiro, Hadassa Loth de Oliveira, Danielly C. O. Mariano, Yasmin Salazar Torres, Graciela Maria Dias, Bianca C. Neves

**Affiliations:** ^1^ Instituto de Química Universidade Federal do Rio de Janeiro Rio de Janeiro Brazil

**Keywords:** adaptation, *Burkholderia cenocepacia*, comparative Genomics, phylogeny, type III secretion system

## Abstract

Type III secretion systems (T3SS) have been described in many Gram‐negative bacteria, including the human opportunistic pathogen *Burkholderia cenocepacia*, originally described in association with plants. The present work aimed to identify T3SS genes in a group of genomes of isolates from different sources, to gain insight into their role in the life cycle and host association of *B. cenocepacia*. Comparative and phylogenetic analyses were used to investigate the distribution, evolution, variability, and synteny of the T3SS genes within 48 genomes of *B. cenocepacia*. Only one genome, from a human clinical isolate, lacked T3SS genes. These results revealed 9 different clusters of T3SS genes within *B. cenocepacia*, all lacking an annotated needle protein gene (*sctF*) within the T3SS clusters. Interestingly, a CDS encoding a putative SctF was found outside the main T3SS gene clusters, which is highly conserved in all analyzed genomes. Taken together, these results suggest that the T3SS gene clusters seem to have been independently acquired and may play a pivotal role in pathogenicity, host range determination, and niche adaptation.

## Graphical abstract

T3SS is a dedicated machinery that mediates interactions between bacteria and eukaryotic cells, providing a functional interface in pathogenic or symbiotic associations. Phylogenetic analyses and T3SS‐gene synteny revealed nine gene clusters, with no needle protein gene (*sctF*). However, an orphan CDS encoding a putative SctF was found.

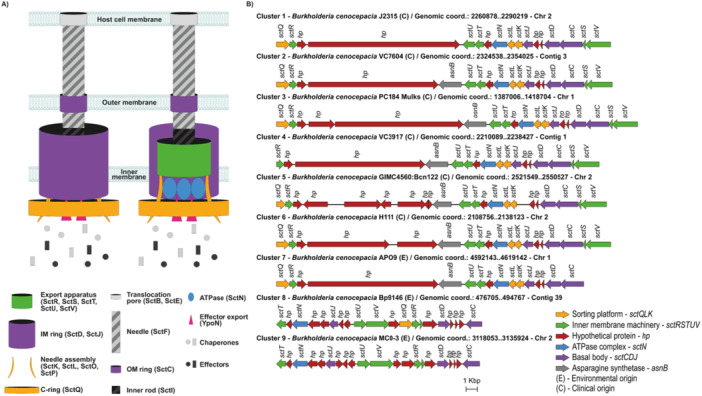

## 1 Introduction

The genus *Burkholderia* comprises Gram‐negative non‐fermenting bacilli with widespread environmental occurrence, colonizing a variety of ecological niches (Coenye and Vandamme 2003). The genus taxonomy has been subjected to many revisions in the past decade, resulting in the proposal of six novel genera: *Burkholderia* sensu stricto (s.s.), *Paraburkholderia*, *Caballeronia*, *Trinickia*, *Mycetohabitans* and *Robbsia* (Dobritsa and Samadpour 2016; Estrada‐de los Santos et al. 2018; Lopes‐Santos et al. 2017; Sawana et al. 2014). The *Burkholderia cepacia* complex (BCC) is a group of closely related species, widespread in the environment and commonly associated with plants (Vandamme and Dawyndt 2011). BCC members are human opportunistic pathogens and are a cause of great concern for cystic fibrosis (CF) patients, capable of developing chronic, hard‐to‐treat lung infections (Drevinek and Mahenthiralingam 2010; Scoffone et al. 2017). BCC lung infections can evolve into a systemic condition called Cepacia Syndrome, with increased mortality rates. *B. cenocepacia* is one of the most prevalent agents within BCC opportunistic infections and has been widely used as a model (Drevinek and Mahenthiralingam 2010; Scoffone et al. 2017). In molecular epidemiology studies, the species is divided into four *recA*‐based phylogenetic subgroups, with subgroup IIIA containing the lineage ET12, usually associated to Cepacia Syndrome. Some *B. cenocepacia* subgroups can be frequently isolated from natural environments and in association with plants, including subgroup IIIA (Baldwin et al. 2007; Dalmastri et al. 2007; Vandamme et al. 2003).

In addition to their clinical importance, many *B. cenocepacia* isolates can colonize plant endosphere and rizosphere, either causing disease (Jacobs et al. 2008) or playing a protective role against plant pathogens (Ho et al. 2015; Rojas‐Rojas et al. 2019; Song et al. 2024; Tagele et al. 2019). Mechanistically, in pathogenic contexts, a functional T3SS is often required to suppress host immune responses and enable successful colonization (Horna and Ruiz 2021; Su et al. 2025). Conversely, beneficial *B. cenocepacia* isolates have been shown to promote plant growth, trigger systemic resistance, and protect against phytopathogens (Stringlis et al. 2019; Zboralski et al. 2022), illustrating the ecological versatility and context‐dependent outcomes of these interactions. In either way, plants may represent important reservoirs for *B. cenocepacia* in the environment, as intermediate or alternative hosts. However, the molecular interface mediating *B. cenocepacia*‐plant interactions remains to be investigated.

Bacteria have evolved specialized molecular mechanisms of adaptation to their environments, which include interaction with eukaryotic hosts and members of microbial communities (Christie 2019). Bacterial secretion systems are in the frontline of these molecular interfaces, mediating cross‐talks with eukaryotic hosts and playing a pivotal role in the outcome of pathogenic or symbiotic relationships. Some of them, including type III (T3SS), type IV (T4SS), and type VI (T6SS) secretion systems, have been extensively studied in bacteria‐host interactions (Christie 2019; Costa et al. 2015). While T3SS is dedicated to translocation of effector proteins into eukaryotic host cells, T6SSs are widespread, versatile machineries, which deliver effector proteins into eukaryotic host cells or microbial competitors and predators in their environment (Christie 2019; Costa et al. 2015; Notti and Stebbins 2016). T3SSs have been described as mediators of pathogenic (Büttner and He 2009; Schmidt et al. 2012) or mutualistic (Tampakaki 2014) interactions with a wide variety of hosts by establishing a trans‐kingdom communication channel (Notti and Stebbins 2016). In the case of plants, T3SSs are used to evade the host's immune system and colonize the inner tissues.

A T3SS locus has been identified in a clinical isolate of *B. cenocepacia* and is conserved among clinical and environmental isolates (Tomich et al. 2003). However, studies showing the molecular mechanisms involved in plant associations are yet to be described. Classifying bacteria into plant or human pathogens is a difficult task in some species, especially those with large and plastic genomes such as *B. cenocepacia*, which carry very diverse gene sets, conferring adaptation to a wide range of ecological niches and hosts (Eberl and Vandamme 2016; Etesami 2025). The present study focuses on the genomic identification and characterization of T3SS gene clusters within the genomes of environmental and clinical isolates of *B. cenocepacia*.

## 2 Materials and Methods

### 2.1 Selection and Annotation of Bacterial Genomes

48 genomes of *B. cenocepacia*, publicly available at the time of the study, were selected according to their origin, clinical or environmental, excluding genomes of unknown origin. The database used was the GenBank (Benson et al. 2014), a database from the National Institutes of Health (NIH). Parameters for selection included genome completeness, number of contigs or scaffolds below 200. In addition, sequencing methods by Illumina and/or PacBio were also prioritized. The functional annotation of the selected genomes was performed using Prokka (v. 1.14.6) (Seemann 2014).

### 2.2 Comparative Genomic Analysis

Comparative genomic analysis was carried out by mapping against the database T3Enc (Hu et al. 2017). To identify T3SS genes in the selected genomes, the BLASTp tool (Altschul et al. 1990) was used, with the parameters of 40% similarity and e‐value of 10E‐3. The best hits were filtered and organized for further analysis. The Easyfig visualization tool (v. 2.2.2) (Sullivan et al. 2011) was used to show the T3SS gene clusters present in the genomes. Files with the.gbk extension were obtained from genomic annotations and used as input.

### 2.3 Phylogenetic Analyses

For taxonomic and phylogenetic analyses of the *B. cenocepacia* genomes, *recA* gene was initially employed, followed by a multilocus sequence analysis (MLSA) based on seven housekeeping genes—*atpD* (ATP synthase beta 1subunit), *gltB* (ferredoxin‐dependent glutamate synthase 1), *gyrB* (DNA gyrase subunit B), *recA* (protein RecA), *lepA* (elongation factor 4), *phaC* (poly (3‐hydroxyalkanoate) polymerase subunit PhaC), and *trpB* (tryptophan synthase beta chain). Finally, *sctN*, which encodes a T3SS‐associated ATPase, was employed to assess phylogeny within the T3SS. The multiple gene alignments were performed with the software Clustal Omega (Sievers et al. 2011). Alignments were calculated through the Hhalign package (Söding 2005), which aligns two Markov models of hidden profiles. On the Clustal Omega platform, ClustalW was used as the output format. After the alignment, the phylogenetic analyses were conducted with the MEGA X software (Kumar et al. 2018), with a set of tools to infer evolutionary trees, estimate genetic distances, and reconstruct ancestral sequences. The Maximum Likelihood method, with a bootstrap value of 1,500, allowed the determination of similarities and differences between genomes and their origins. This method made it possible to estimate lower variance, with fewer errors of sampling (Caldart et al. 2018). Nucleotide‐substitution models were Tamura‐Nei (*recA*), General Time Reversible (MLSA), and Tamura 3‐parameter (*sctN*) (Tamura 1992; Tamura et al. 2011). The phylogenetic trees were visualized with the online platform iTOL (v. 5) (Letunic and Bork 2021).

### 2.4 Taxonomic Analysis

The Average Nucleotide Identity (ANI) and DNA‐DNA digital hybridization (dDDH) were used for taxonomic analyses and to confirm whether the genomes belonged to the same species (Auch et al. 2010), respectively. PyANI version 0.2.10 (https://github.com/widdowquinn/pyani/tree/master) is a tool designed for whole‐genome microbial classification based on ANI and was used in this study, as it is a tool specifically designed for this purpose. The strains that shared more than 95% identity (ANI), covering at least 70% of the genome sequence, were regarded as the same species. dDDH values of the analyzed genomes were obtained through the web service Type (Strain) Genome Server (TYGS), at (https://tygs.dsmz.de) (Meier‐Kolthoff and Göker 2019) using dDDH‐d4. Values below 70% were considered as an indication that the tested genomes were from different species (Auch et al. 2010), in which case they were discarded. For the ANI and dDDH taxonomic analyses, heatmaps of their respective values were used, with the matplotlib and seaborn Python plotting library (v. 3.9.2) (https://github.com/matplotlib/matplotlib). The distance and clustering methods were Euclidean and average, respectively.

### 2.5 Prospection of a Needle Protein Gene—*sctF*

Initially, STEPdb (https://stepdb.eu/strains/epec/homologues) (Orfanoudaki and Economou 2014) was used to prospect homologous sequences related to the SctF (needle protein) in the genomes of *B. cenocepacia*. The reference SctF proteins used for the preliminary prospection were from *Bordetella*, *Pseudomonas syringae*, *Ralstonia solanacearum*, *Xanthomonas* spp., and *Burkholderia pseudomallei*, which are plant‐associated species. The BLAST (version 2.16.0) command‐line tool was used to build the database with homologous sequences, followed by alignment with the annotated CDS of each *B. cenocepacia* genome, performed with blastp (version 2.16.0) and e‐value 10E‐5. Finally, the putative SctF sequence from *B. cenocepacia* was aligned with its structural homologs, including PrgI from *Salmonella enterica* subsp. *enterica* serovar *Typhimurium* (Protein Data Bank, 2LPZ), BsaL from *B. pseudomallei* (Protein Data Bank, 2G0U), and PscF from *Pseudomonas aeruginosa* PA14 (*Pseudomonas* Genome Database, PA14_42310).

Multiple sequence alignment (MSA) was performed using MAFFT (version 7.490 dated 10/30/2021) (Katoh et al. 2002), employing the E‐INS‐i option with the ‐‐genafpair and‐‐ maxiterate 3000 parameters. Alignment visualization and secondary structure representation were performed using ESPript 3.0 (Robert and Gouet 2014), with the 2LPZ reference structure, and the sequence similarities depiction parameters were %Equivalent and Global Score set to 0.7.

### 2.6 Homology Modeling of SctF

Rosetta‐based helical modeling was employed, with the protocol outlined by DiMaio et al. (2011). The alignment of the putative SctF from *B. cenocepacia* was performed with the atomic model of PrgI, the needle protein of *Salmonella* (*Salmonella enterica* subsp. *enterica* serovar Typhimurium), derived from solid‐state NMR and available in the Protein Data Bank (PDB ID: 2LPZ). PrgI served both as a template for homology modeling and as a reference for helical symmetry. This approach resulted in several distinct clusters of decoys. The lowest‐energy decoys that closely resembled the PrgI needle architecture were prioritized in detriment of alternative clusters with implausible conformations.

### 2.7 Statistical Analyses

All analyses were performed using Student's *t*‐test in GraphPad Prism version 8.0.0 (GraphPad Software, San Diego, CA, USA, www.graphpad.com). Results with a *p*‐value ≤ 0.05 were regarded as statistically significant.

## 3 Results

### 3.1 Genome Selection and Annotation

During this study, there were 329 genomes of *B. cenocepacia* in the GenBank, comprising drafts and complete genomes. Among all listed genomes, a filter was applied to select strains of clinical or environmental origin. Genomes of selected strains from environmental origin were isolated from different sources—soil, aerosols, water, rhizosphere, and corn root. The selected strains of clinical origin were from blood, sputum, cord blood, and nasal sources. A total of 48 genomes were selected and annotated, initially revealing transfer RNAs (tRNA), transfer‐messenger RNA (tmRNA), and CRISPR RNA. All 48 genomes have a Guanine‐Cytosine content (GC%) higher than 60%, tending to have high numbers of tRNA genes, larger genome sizes, and high predicted rates of cell growth, as expected for prokaryotes (Satapathy et al. 2010). These data are presented in Supporting Information S1: Table S1 for strains of environmental origin and in Supporting Information S1: Table S2 for strains of clinical origin, along with the geographic coordinates of the sites where the strains were isolated. Taken together, their geographical distribution is shown in Supporting Information S1: Figure S1.

### 3.2 Comparative Analyses and Genomic Organization

For a more comprehensive view, the variability of genomic organization and the whole‐genome average sizes (Mb) were assessed and compared, along with coding sequences (CDS), and GC content (GC%) for the strains of environmental or clinical origin. Supporting Information S1: Figure S2 shows that strains of environmental origin had an average size of 7.65 Mb, while the clinical strains exhibited an average size of 7.77 Mb, demonstrating no significant difference (Student *t*‐test, *p* < 3.55E‐01). The average number of CDS was 6803 and 6981, for strains of environmental or clinical origin, respectively, indicating that there is no significant difference (Student *t*‐test, *p* < 1.68E‐01) (Supporting Information S1: Figure S3). Regarding the GC content, the average GC% for environmental genomes was 66.85% and for the clinical samples 66.99%, showing no significant difference (Student *t*‐test, *p* < 1.14E‐01; Supporting Information S1: Figure S4).

### 3.3 Syntenic Organization of the T3SS Genes

**Figure 1:**
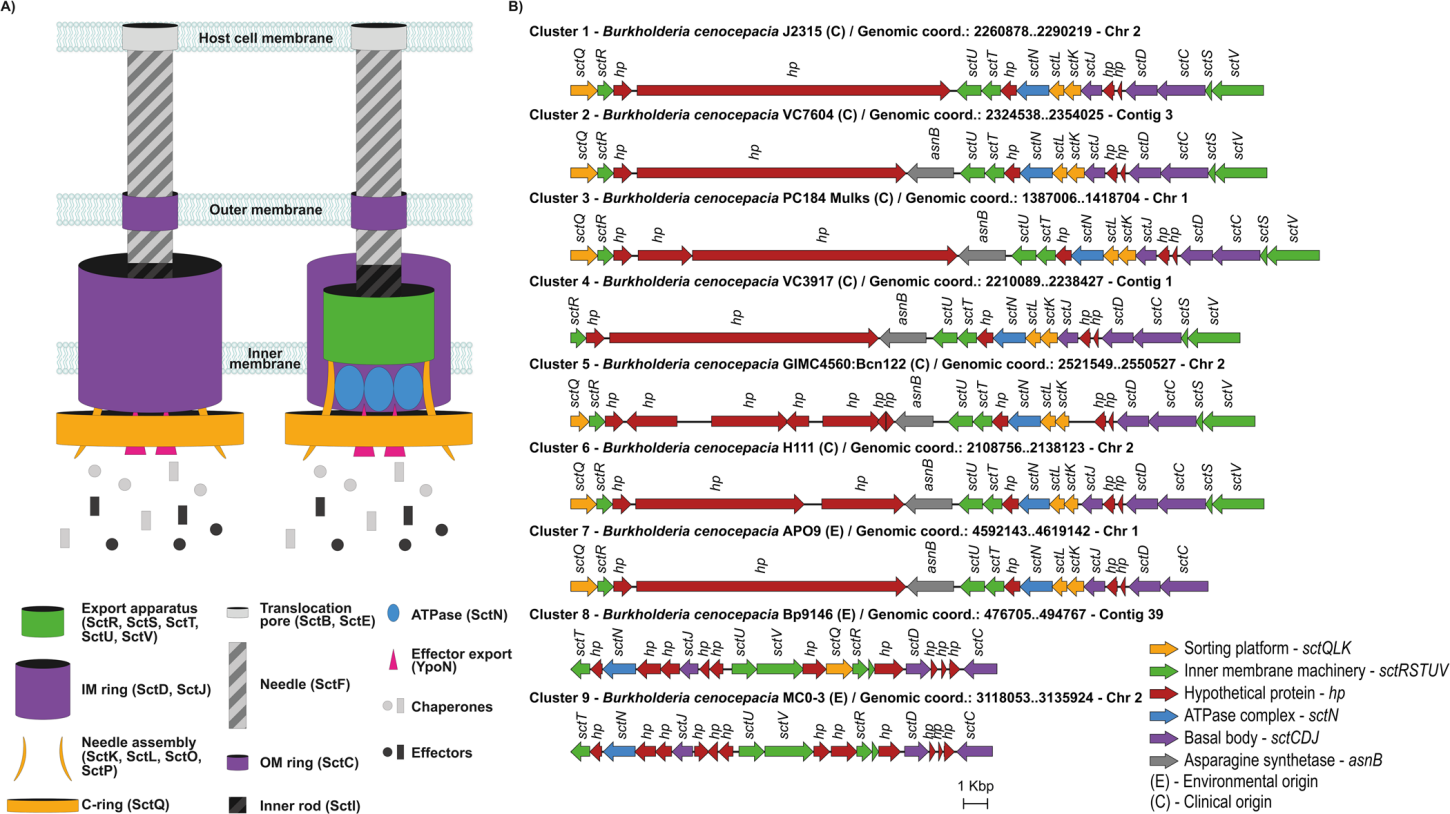
Depiction of a Type III Secretion apparatus and its coding gene clusters in *B. cenocepacia*. (A) The diagram illustrates the main structural components, including cytosolic, transmembrane, and extracellular components. (B) Representation of the nine gene clusters described in this study and annotation of the known encoded components.

Nine distinct clusters of T3SS were identified within the genomes, as shown in Figure 1, with genes that encode structural components (sct) in all but one genome. 38 genomes contain only one cluster, nine have two clusters, and 1 has no T3SS genes. Strains that present only one T3SS cluster are either of environmental or clinical origin, while those that have two clusters are of environmental origin only. The only strain that does not present any T3SS cluster (*B. cenocepacia* VC9789) is from a human clinical origin (sputum).

Interestingly, the *asnB* gene, which encodes an asparagine synthetase, maps within T3SS clusters 2, 3, 4, 5, 6, and 7 (Figure 1). AsnB is defined as a virulence‐related factor, regulated by both the diffusible signal factor (DSF) and the global regulator cAMP Receptor‐Like Protein (Clp) in *Xanthomonas oryzae*, and highly conserved in *Xanthomonas* (Qian et al. 2013). Another putative gene, identified in clusters 1, 2, 3, and 4, attracts attention for its length (6,637 bp) and encodes a hypothetical protein (GenBank accession: WP_321175898—*B. cenocepacia* J2315) (Figure 1). A Blast search revealed three main domains within this protein: HopBF1, DUF4781, and SEC. 10/PgrA Surface Exclusion Domain, with e‐values of 3.19E‐34, 8.00E‐10, and 2.90E‐09, respectively, indicating high reliability. The HopBF1 domain is widespread in plant pathogens, playing a role in the inactivation of an essential chaperone (Heat Shock Protein—HSP90) necessary for the innate immunity in plant hosts, inducing necrosis and collapse in plant leaf tissues (Lopez et al. 2019). The DUF4781 domain, despite being widely found in eukaryotes and bacteria, has not been functionally characterized (Bateman et al. 2010). The SEC. 10/PgrA Surface Exclusion Domain is conserved in bacteria, usually found on cell surface proteins of various Firmicutes, involved in the specific inhibition of the bacterial cell's ability to acquire homologous plasmids, a process known as “surface exclusion” (Kao et al. 1991). While the potential role of this protein (WP_321175898) in plant‐associated interactions remains speculative in *B. cenocepacia*, the presence of these domains suggests it could be a target for future functional investigations related to host‐pathogen interactions.

The length and the content of the identified T3SS clusters are variable, with some missing structural genetic elements (Figure 1). Cluster 4, which is present in six *B. cenocepacia* genomes of clinical origin, lacks gene *sctQ*. Cluster 5 is present in only one genome, GIMC4560:Bcn122, of clinical origin, and does not have the *sctJ* gene. Cluster 8, present in nine strains of environmental origin, does not harbor the *sctKL* genes; cluster 9, found in the environmental strain MC0‐3, lacks the *sctKLQ* genes. *sctJ* encodes a structural T3SS protein (SctJ), which forms the inner‐membrane ring of the apparatus, controlling protein transport through the system (Pais et al. 2023). The *sctKLQ* genes encode protein components involved in the hierarchical recruitment of secreted substrates of the T3SS apparatus. Cluster 7, which exclusively encompasses the APO9 strain of *B. cenocepacia*, is not a complete cluster because it lacks the *sctSV* genes. These genes are essential for encoding an export apparatus, with SctS acting as the entry point for the secretion channel, while SctV functions as a proton/protein antiporter. Functional categories, structural or secreted components, play pivotal roles, and their absence would completely impair the secretion process. However, the occurrence of those missing components, such as cognate orphan T3SS genes, might potentially complement these incomplete apparatuses.

**Figure 2:**
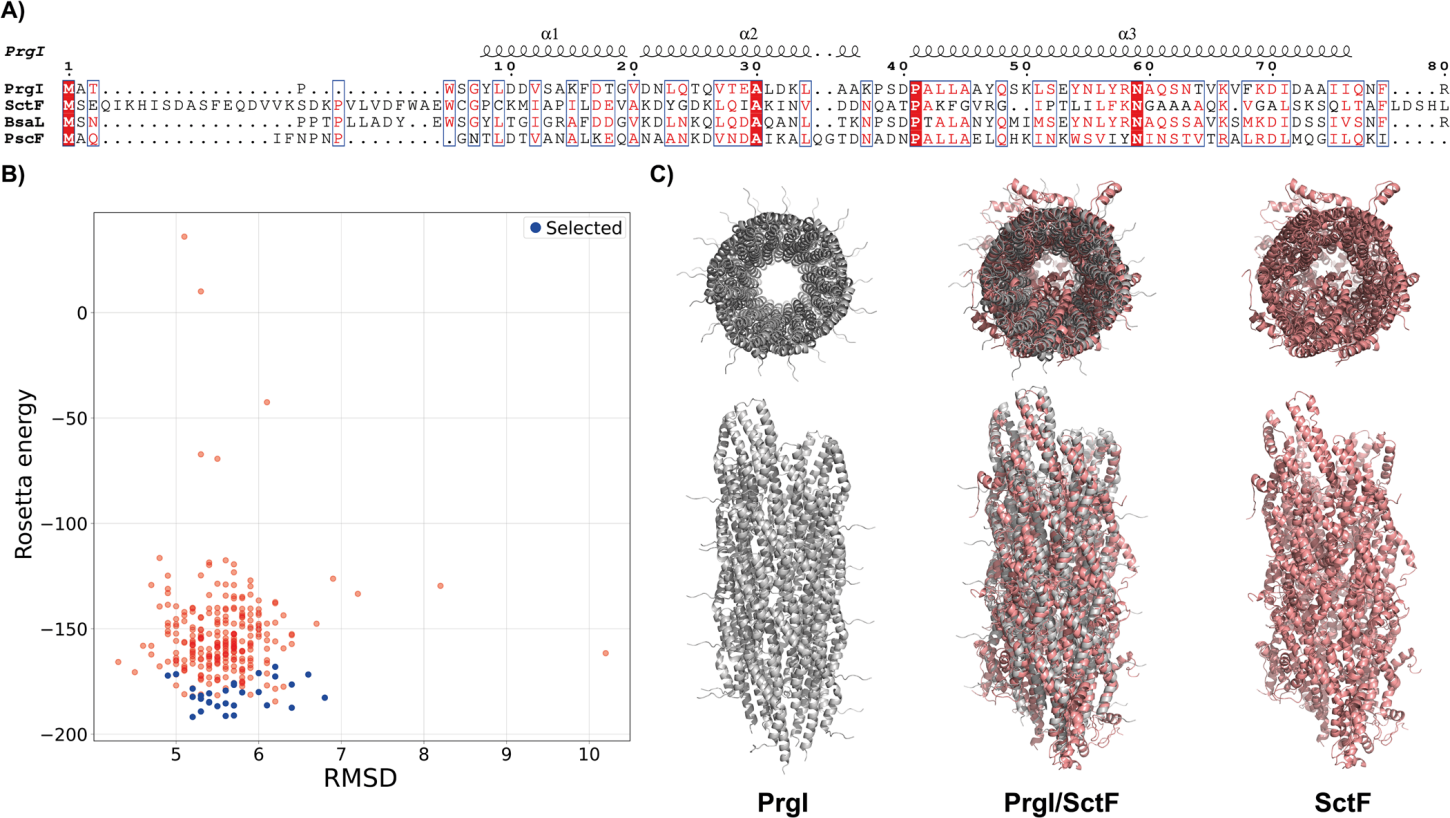
*In silico* modeling of a putative needle protein of *B. cenocepacia*. (A) Comparative analyses of the putative SctF sequence from *B. cenocepacia* and its functional analogs, including PrgI from *S. enterica* subsp. *enterica* serovar *Typhimurium* (Protein Data Bank, 2LPZ), BsaL from *B. pseudomallei* (Protein Data Bank, 2G0U), and PscF from *P. aeruginosa* PA14 (*Pseudomonas* Genome Database, PA14_42310). The multiple alignment shows the conserved residues highlighted in a solid red box, while similar residues are shown in red type. The putative SctF needle protein exhibits a secondary structure similar to experimentally defined needle proteins (e.g., PrgI), with three major α‐helices. (B) Results of the SctF symmetry homology modeling obtained with Rosetta. The energy of each decoy model is plotted against the root mean square deviation values. The backbones of the lowest energy models are shown in blue and were used in the following model. (C) The PrgI NMR‐derived tridimensional needle structure, with 29 identical protein subunits (Protein Data Bank, 2LPZ) was employed for a comparative analysis against the *in silico* SctF needle model obtained with Rosetta. The PrgI needle was used as template (light gray), matching the subunits of the modeled SctF needle (light red).

Interestingly, further comparative genomic analyses revealed the presence of a conserved CDS in all genomes, including the strain lacking other T3SS genes, mapping outside the main T3SS clusters and coding for a putative SctF, the “needle” protein. Although a canonical needle protein is not available, predicted α‐helix structures suggest this CDS could potentially encode an SctF analog (Figure 2, Supporting Information S1: Table S3 and Figure S5). Remarkably, the amino acid sequence of the putative SctF was identical across all strains analyzed in the present study (Supporting Information S1: Figure S5). For a comparative analysis, the sequence was aligned with its homologs, including PrgI from *S. enterica* subsp. *enterica* serovar *Typhimurium* (Protein Data Bank, 2LPZ), BsaL from *B. pseudomallei* (Protein Data Bank, 2G0U), and PscF from *P. aeruginosa* PA14 (Pseudomonas Genome Database, PA14_42310) (Figure 2).

### 3.4 Phylogenetic Analysis Based on the *sctN* Gene

**Figure 3:**
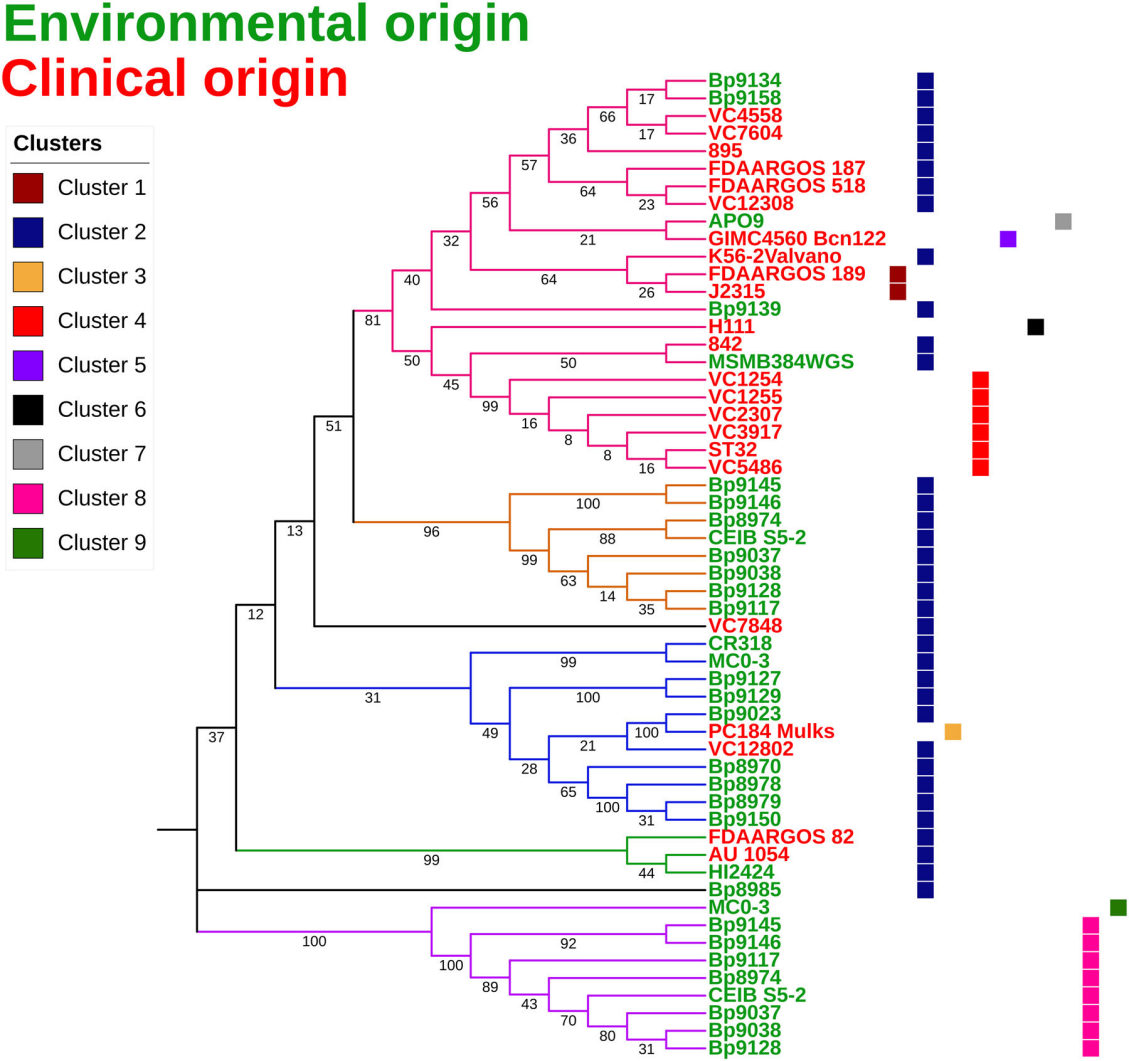
Phylogeny based on the *sctN* of T3SS. Five major clades were generated, designated I to V. Clade I (pink) comprises mainly clinical strains, while Clade II (yellow) is composed exclusively of environmental isolates. All genomes in Clade II contain cluster 2 of the T3SS. Clade III (blue) contains mainly environmental genomes, and the cluster 2. Clade IV (green) contains 2 clinical and 1 environmental genome, all of which have only the cluster 2. Clade V (violet) contains only environmental genomes, with cluster 8 being the most frequent. Clinical strains are shown in red, while environmental ones are in green. Genomes VC7848 (clinical) and Bp8985 (environmental) did not group in any clade, despite sharing cluster 2 of T3SS.

The *sctN* is an essential core gene that encodes an ATPase within the T3SS apparatus and is one of the most conserved and frequently used T3SS core genes for phylogenetic analysis in several studies (Teulet et al. 2020). To better understand the importance and distribution of T3SS in *B. cenocepacia*, we selected the *sctN* gene from the analyzed genomes as an input to obtain the phylogenetic relationships and placement of strains based on their T3SS cluster (Figure 3). The first clade (clade I), containing mainly clinical strains (18 strains), also included five strains of environmental origin (Bp9134, Bp9158, APO9, Bp9139, and MSMB384WGS). The T3SS clusters identified in this clade are 1, 2, 4, 5, 6, and 7. The *sctN* of the five environmental strains of this clade belongs to T3SS cluster 2 and has an identity of > 95% with the *sctN* of other *Burkholderia* species, such as the type strain *Burkholderia metallica* AU0553^T^ ( = LMG 24068 ^T^ = CCUG 54567 ^T^), isolated from a CF patient and a member of the BCC (Vanlaere et al. 2008). The second clade is composed of strains of environmental origin, all presenting the T3SS cluster 2. Notably, all strains in this clade are of environmental origin and harbor two distinct T3SS clusters, which also group them into the fifth clade (clade V), along with the MC0‐3 strain, which is also of environmental origin. The third clade predominantly includes strains of environmental origin, along with two clinical strains, PC184 Mulks and VC12802. Most of the strains in this clade harbor the T3SS cluster 2, except for the clinical strain PC184 Mulks, which stands out for presenting cluster 3, an exclusive cluster to this strain. Clade IV includes only three strains, two of clinical origin (FDAARGOS 82 and AU 1054) and one of environmental origin (HI2424), all presenting T3SS cluster 2. Clade V comprises the *sctN* gene from clusters 8 and 9, with cluster 9 identified exclusively in the MC0‐3 strain. In the second clade (clade II) most strains are of environmental origin and harbor T3SS cluster 2, while five of them are of clinical origin, one of which contains cluster 3.

### 3.5 Genetic Taxonomy

**Figure 4:**
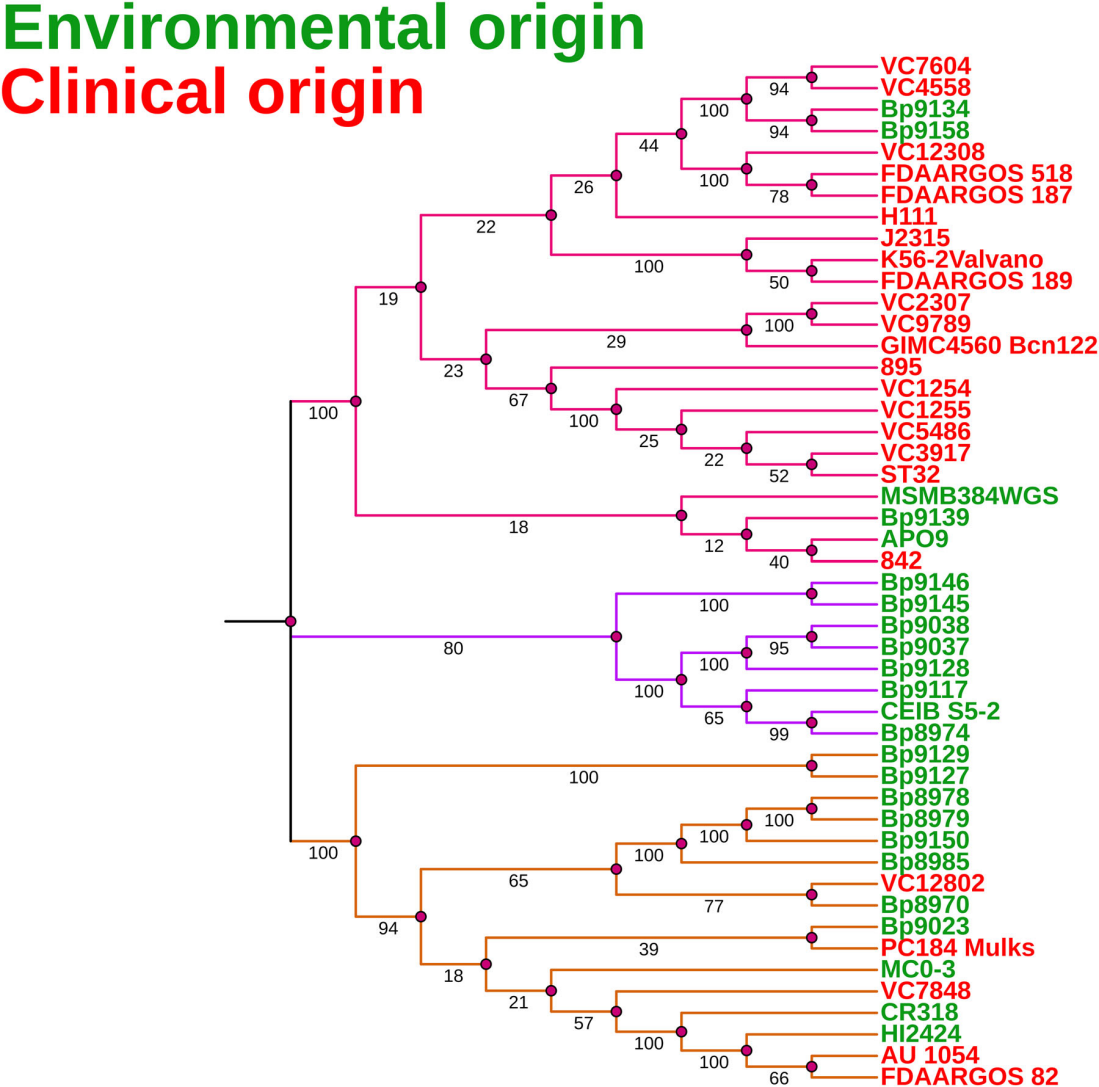
Housekeeping‐based phylogenetic analysis. Three clades have been defined when housekeeping genes were employed (*recA*, *gyrB*, *atpD*, *gltB*, *lepA*, *phaC*, and *trpB*). Clade I (pink) comprises mainly clinical isolates, clade II (purple) includes only strains of environmental origin. Clade III (yellow) contains mainly environmental strains.

Another taxonomic approach to analyzing *B. cenocepacia* strains is MLSA, covering internal fragments of several protein‐coding genes (housekeeping genes), which evolve constantly and at a slower rate than the traditional 16S rRNA gene (Glaeser and Kämpfer 2015), providing better discrimination at the genus or lower taxonomic levels. It is believed that MLSA‐based trees reflect a more reliable relationship between bacterial taxa (Ansari et al. 2019; Estrada‐De Los Santos et al. 2013). The MLSA method is widely used (Ansari et al. 2019; Baia et al. 2021; Estrada‐De Los Santos et al. 2013; Gautam et al. 2016; Jin et al. 2020; Peeters et al. 2013) due to its high discriminatory power for differentiating BCC species (Baia et al. 2021). The housekeeping genes *recA*, *gyrB*, *atpD*, *gltB*, *lepA*, *phaC*, and *trpB* were used as the basis for constructing a taxonomic tree with three distinct clades (Figure 4), with high bootstrap values, allowing significant distinction between the lineages.

The first clade (clade I) predominantly comprises clinical strains, except for four strains of environmental origin (Bp9139, MSMB384WGS, Bp9134, and Bp9158). Interestingly, further genome prospection revealed these environmental strains harbor virulence genes. Specifically, the environmental strain MSMB384WGS carries genes involved in virulence, such as *22 kDa adhesion* (*adhA*), *transcriptional regulator kdgR* (*kdgR*), *bile acid 7‐alpha dehydratase* (*baiE*), *taurine dehydrogenase* (*tauX*), *sulfoacetaldehyde acetyltransferase* (*xsc*), *tellurite resistance cluster* (*telA*), *nitrate sensor and regulation cluster* (*narLX*), and the *respiratory nitrate reductase cluster* (*narIJHGK*) (Wallner et al. 2019). Additionally, the environmental strains Bp9139, Bp9134, and Bp9158, analyzed solely in the present study, were found to possess *kdgR*, *baiE*, *xsc*, and *narIJHGK* virulence genes. Moreover, the environmental strain APO9, also analyzed in our study, was found to harbor *kdgR*, *telA*, and *narJ*. The second clade (clade II) is comprised only by strains of environmental origin. The third clade (clade III) comprises strains of environmental origin, except for five strains (VC7848, AU 1054, FDAARGOS 82, VC12802, and PC184 Mulks) of clinical origin. Four of these strains (VC7848, AU 1054, VC12802, and PC184 Mulks) were also grouped into clades encompassing environmental strains in the tree constructed based on the *sctN* gene and are known to carry genes that are involved in bacteria‐plant association. According to Wallner et al. (2019), strain VC7848 carries *nitrile hydratase subunits alpha and beta* (*nthAB*), *phenylacetaldoxime dehydratase* (*oxd*), *feruloyl‐esterase* (*faeB*), and *galacturonate metabolism genes* (*uxaAB*); strain AU 1054 contains *lectin‐like bacteriocin 88* (*llpA*), *nthAB*, *oxd*, *faeB*, and *uxaAB*; strain VC12802 possesses *llpA*, *nthAB*, *oxd*, *faeB*, *pyrrolnitrin biosynthesis cluster* (*prn*), and *uxaAB*; and strain PC184 Mulks harbors *nthAB* and *prn*. These genes contribute to plant‐associated fitness by facilitating carbon source conversion into the phytohormone indole‐3‐acetic acid (IAA), thus enhancing plant growth, and by promoting rhizodeposition, which increases microbial biomass in the rhizosphere (Etesami et al. 2015; Howden et al. 2009; Liu et al. 2017; Spaepen et al. 2007; Wallner et al. 2019). Additionally, the clinical strain FDAARGOS 82, which was not investigated in the study by Wallner et al. (2019), was analyzed in the present study for the presence of genes related to virulence or plant‐associated bacterial fitness. I five genes linked to plant‐associated fitness were found: *nthA*, *nthB*, *oxd*, *uxaA*, and *uxaB*. The protein products encoded by *nthAB*, *oxd*, and *faeB* are involved in the conversion of carbon sources such as galacturonic acid, xylans, and pectin into IAA, thereby promoting plant growth. In turn, the proteins encoded by *uxaAB* participate in rhizodeposition processes that enhance microbial biomass in the plant rhizosphere (Lima and da 2014; Suvorova et al. 2011; Wallner et al. 2019).

**Figure 5:**
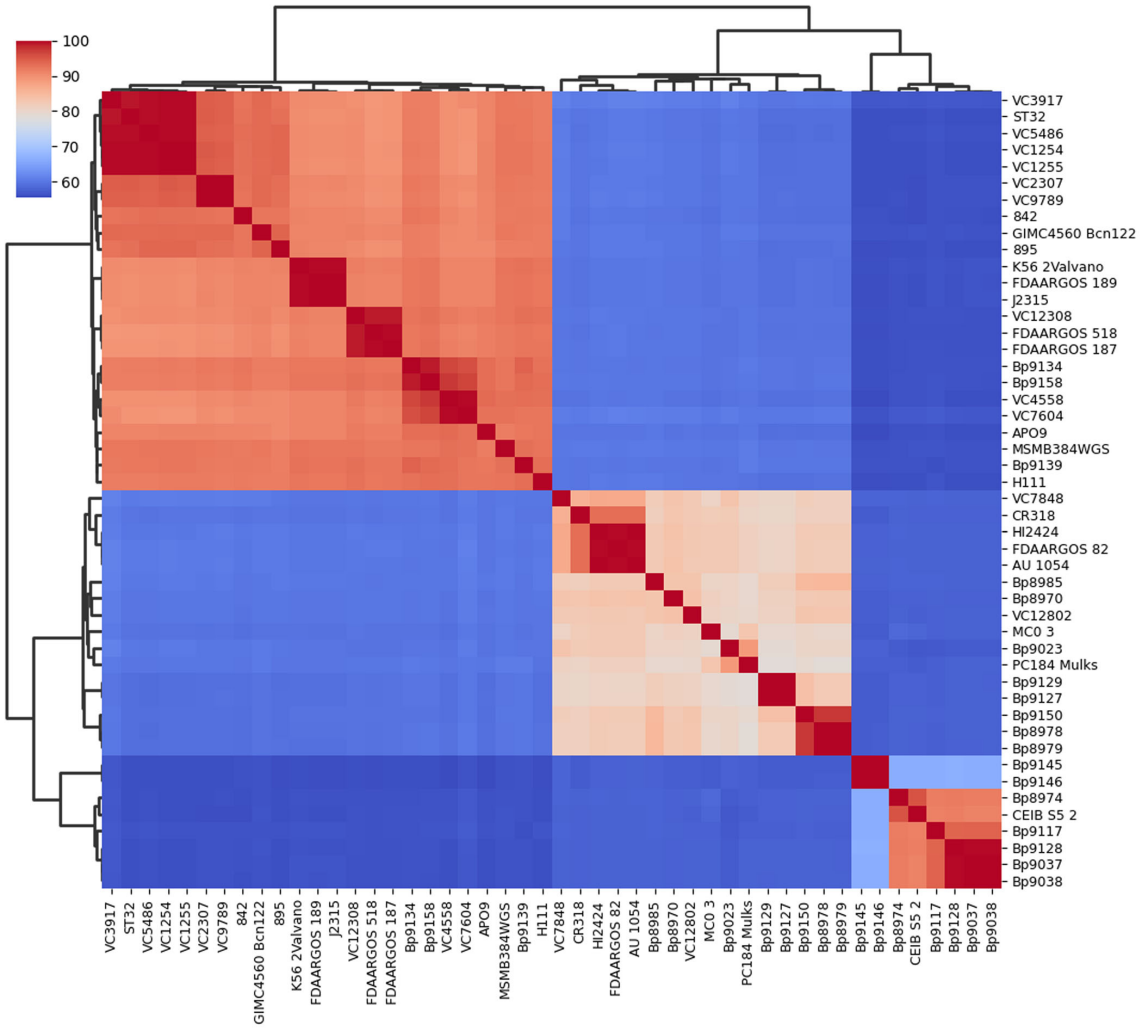
Heatmap of dDDH values. Genome‐to‐genome comparisons yielded three clusters in the heatmap. While the threshold for distinguishing species is > 95%, dDDH values indicate that all strains belong to the same species, *B. cenocepacia*.

**Figure 6:**
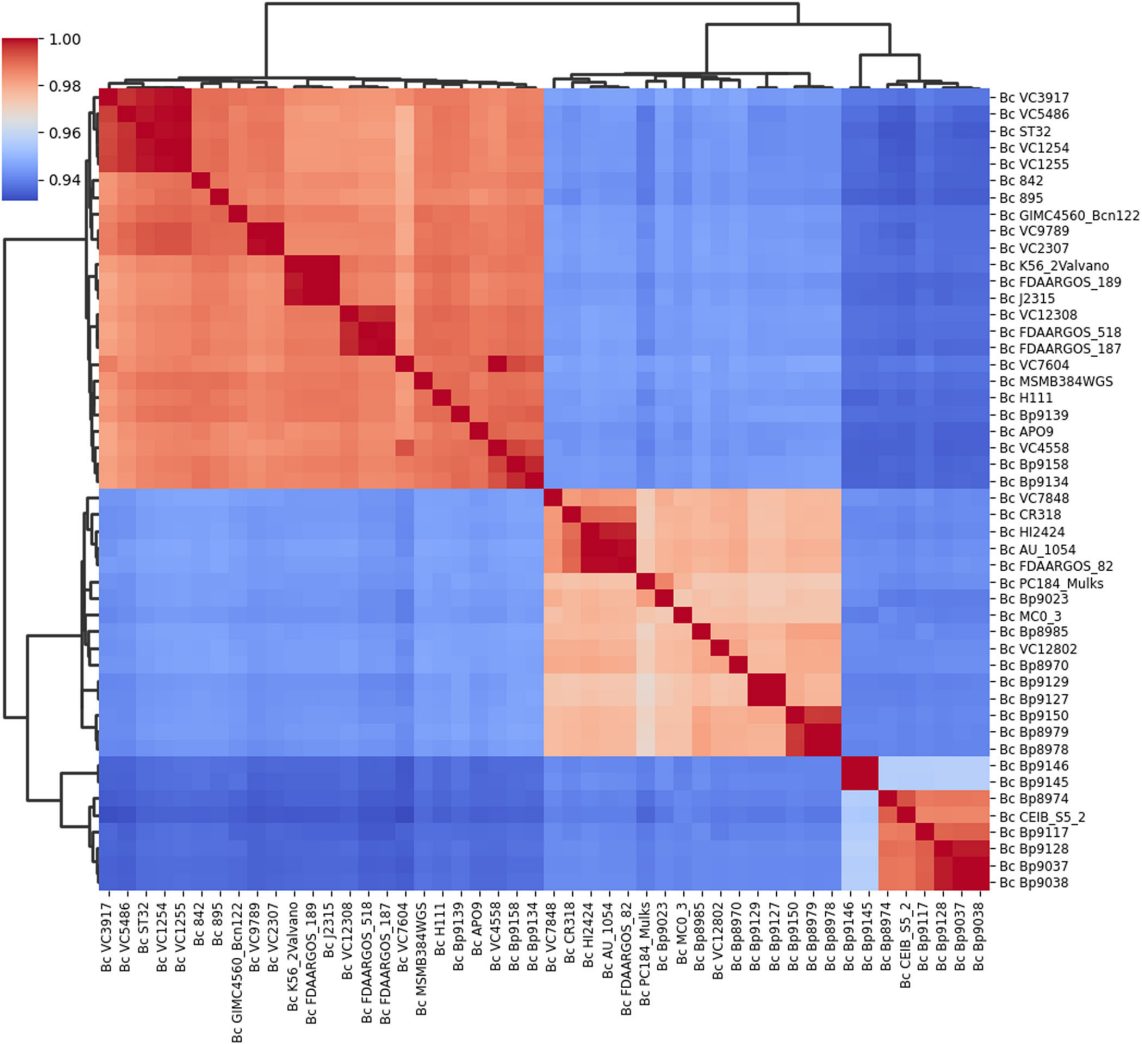
Heatmap of ANI values. The average nucleotide identity yielded three clusters in the heatmap. As the threshold for distinguishing species is > 70%, the values suggest that all genomes belong to the same species, *B. cenocepacia*.

To investigate the general features among the genomes, and further clarify the taxonomic and phylogenetic results, a comparative analysis was carried out with the dDDH and ANI values, as shown in Figures 5 and 6, respectively. Observing both heatmaps (dDDH and ANI) there are three main clades. In the taxonomic heatmaps, two major clades were identified, with the second clade subdivided into two subclades. Remarkably, these subclades display two identical groups of strains when compared to the taxonomic profile obtained in the MLSA tree (Figure 4). The genomes of the strains from the first clade have an ANI between 97.7% and 100% (Figure 6) and share ≥ 89.2% identity in the dDDH values (Figure 5) and are therefore regarded as the same species. Despite the genomes of the second clade (first subclade) having ANI of 96.7% to 100%, we believe they are grouped as a single species, as dDDH values are ≥ 78.6%. The third clade (second subclade) presents genomes with ANI ranges between 95.4% and 100% and dDDH values ≥ 91.3%. Finally, we confirm that the strains grouped in the first, second, and third clade (except strains Bp9145 and Bp9146) belong to the same bacterial species, *B. cenocepacia*. Strains Bp9145 and Bp9146 are grouped in the third clade with other environmental strains. Still, they have low values of dDDH (≥ 66.4%) (Figure 5), which can suggest that these two strains may belong to another species of the genus *Burkholderia*, despite having ≥ 98.5% ANI (Figure 6).

## 4 Discussion

### 4.1 Genomic Features and Distribution of T3SS Genes in *B. cenocepacia*

The present study provided valuable insights into genome annotation, comparative analysis, and organization of T3SS gene clusters, phylogenetic analysis, and genetic taxonomy of *B. cenocepacia*. Pathogenicity and bacterial adaptation capacity are consistently correlated with genome size, demonstrating a direct relationship with adaptation and an inverse relationship with pathogenicity (Murray et al. 2021; Oliveira et al. 2017). Furthermore, the average genome length is usually associated with the average GC content in prokaryotic genomes, where larger genomes usually exhibit a higher percentage of GC (Almpanis et al. 2018). The GC content of bacteria, ranging from 13% to 75%, is frequently related to the number of coding DNA sequences (CDS) (Oliver and Marín 1996; Mahajan and Agashe 2021). Genomic composition represents an important characteristic contributing to the organism's fitness (Lassalle et al. 2015). As demonstrated in Supporting Information S1: Figures S2 and S3, the selected *B. cenocepacia* genomes do not show significant differences in their main genomic features. This underscores the highly adaptive capacity of *B. cenocepacia*, regardless of the strain's origin.

Although *B. cenocepacia* IIIA strains are typically from clinical origin, their isolation from natural environments is on the increase, frequently associated to plants (Bodilis et al. 2018; Bragonzi et al. 2017). Likewise, in the present study virulence factors are widely distributed in isolates of environmental origin, including plant‐associated *B. cenocepacia*, with genes such as *adhA*, *kdgR*, *baiE*, *xsc*, *telA*, *narJ*, and *narIJHGK*. In 2024, Song et al. showed that the environmental strain APO9 demonstrated the ability to antagonize phytopathogenic fungi, possibly through mechanisms involved in toxin translocation, such as the T3SS. These findings stress the potential role of the T3SS in microbial warfare, typically associated with interkingdom interactions, which are widely distributed in strains of environmental origin (Song et al. 2024). Interestingly, one of the clinical isolates in this study does not harbor any of the T3SS genes shown in Figure 1. In fact, recent studies have demonstrated that the T6SS is the major player in interactions of *B. cenocepacia* with the human host (Loutet and Valvano 2010; Monjarás Feria and Valvano 2020; Romero‐Gutiérrez et al. 2020; Spiewak et al. 2019).

*B. cenocepacia* comprises four major *recA* subgroups, with ET12 epidemic strain belonging to subgroup IIIA, commonly associated with Cepacia Syndrome (Bodilis et al. 2018). Several virulence markers are associated with ET12 strains, such as the 22‐kDa adhesin (encoded by *adhA*) (Drevinek and Mahenthiralingam 2010). Although *B. cenocepacia* is generally considered the most problematic BCC species in patients with CF (LiPuma 2010), plant beneficial endophytic *B. cenocepacia* strains have been reported (Ho et al. 2015; Song et al. 2024). Therefore, plants seem to be potential environmental reservoirs for *B. cenocepacia*. However, the molecular mechanisms of interaction of *B. cenocepacia* with plants need further investigation.

The widespread occurrence of T3SS in *B. cenocepacia*, located on pathogenicity islands, has been described by analyzing homology with the conserved *Yersinia enterocolitica* T3SS (Foultier et al. 2002; Glendinning et al. 2004; Tomich et al. 2003). The Bsc T3SS (Sct, in the unified nomenclature) is composed of at least 10 conserved proteins that are structurally resemblant of the flagellar secretion apparatus (Aizawa 2001; Gophna et al. 2003; Tomich et al. 2003).

The core structure of the T3SS comprises approximately sixteen proteins, thirteen of which are highly conserved: SctC, SctD, SctJ, SctK, SctL, SctN, SctO, SctQ, SctR, SctS, SctT, SctU, and SctV (Figure 1). These proteins are functionally organized into subcomponents: the basal body (SctC, SctD, and SctJ), ATPase complex (SctN), ATPase complex support (SctO), sorting platform (SctK, SctL, and SctQ), and inner membrane machinery (SctR, SctS, SctT, SctU, and SctV). In addition to these core structure proteins, the T3SS contains proteins classified as regulatory (SctP and SctW) and translocator (SctA, SctB, and SctE) components (Notti and Stebbins 2016; Wallner et al. 2021; Pais et al. 2023).

Notably, neither of the T3SS gene clusters analyzed in this study contains regulatory or translocator proteins within the main T3SS clusters, such as the genes that code for the SctO and SctF proteins. While SctO is a cytoplasmic component of the injectisome, assisting the hexameric ATPase SctN, SctF forms the helical needle filament (Broz et al. 2007; Hotinger et al. 2021). At least in *Yersinia*, the absence of the SctO protein does not pose an issue, as the presence of SctK, SctL, and SctQ proteins can facilitate the recruitment of chaperone‐substrate complexes (Diepold et al.2017, Zhang et al.2017) and support SctN, allowing the T3SS apparatus to be assembled and remain functional. Moreover, some Gram‐negative bacteria, commonly associated with plants (*P. syringae* and *Rhizobium* sp.), can constitutively express and retain a functional T3SS, despite the lack of the *sctF* gene within the main T3SS clusters (Gazi et al. 2012; Vander Broek and Stevens 2017). It can be hypothesized that orphan *sctF* genes, located outside these main clusters, could perform a substitutive function. Remarkably, additional genomic analyses in the present study revealed the presence of a highly conserved CDS coding for a putative needle protein (SctF) in all genomes, mapping outside the main T3SS clusters (Supporting Information S1: Table S3, Figure 2). Although a canonical needle protein is not available as a reference, *in silico* studies provided secondary and tertiary structures that strongly suggest the conserved CDS product could be an analog of known, experimentally characterized SctF homologs (Figure 2). However, only a comprehensive, integrative experimental approach combining molecular biology, cryo‐EM, solid‐state nuclear magnetic resonance spectroscopy (NMR), and computational modeling could provide a definite *B. cenocepacia* needle filament and its association with the CDS analyzed in the present study.

### 4.2 Plants as Alternative Hosts for Human and Animal Pathogens

Members of the genus *Burkholderia*, such as *B. cenocepacia*, the focus of this study, have a remarkable ability to adapt to various niches, ranging from environments such as soil and water to infected human patients (Coenye and Vandamme 2003). Likewise, many human and animal pathogens have part of their life cycle outside the human host. These pathogens can adapt to a range of different environments, where the chemical, physical, and biological components are often the “key” to their success. Many well‐known pathogens can move between hosts of different biological kingdoms, being transmitted by the fecal‐oral route or even by direct contact. Many of these, for example, are important enteropathogens. Typical examples are the highly publicized outbreaks caused by *E. coli* O157:H7 and *Salmonella enterica*, which are transmitted by the consumption of food products such as fresh vegetables. These enteric bacteria can actively interact with alternative hosts, such as plants, which are capable of proliferating and maintaining viability (Erickson et al. 2010; Fink et al. 2012; Gu et al. 2013; Saldaña et al. 2011; Zheng et al. 2013). In a similar fashion, five clinical strains from clade II of the *sctN* phylogenetic tree are clustered with environmental strains, not only in this analysis but also in phylogenetic studies employing housekeeping genes (MLSA), dDDH, and ANI (Figures 4, 5, 6). This clustering indicates that the strains VC7848, AU 1054, FDAARGOS 82, VC12802, and PC184 Mulks, despite their clinical origin, possess genomic characteristics typical of strains derived from the environment. Several studies demonstrate that *B. cenocepacia* from environmental sources exhibit the genetic capacity to establish a pathogenic or symbiotic association with multiple hosts, ranging from different plant species to animals (Loutet and Valvano 2010; Springman et al. 2009; Uehlinger et al. 2009). The present study shows strong evidence suggesting that the T3SS may play a pivotal role in the association of *B. cenocepacia* and its non‐human hosts, especially plants, which may serve as environmental reservoirs of this opportunistic pathogen.

## 5 Conclusion

Comparative genomics within the same species of bacteria serves as a valuable approach for the rapid identification of genes associated with host specificity. The T3SS is a dedicated machinery that mediates interactions between bacteria and eukaryotic hosts, providing a functional interface in pathogenic or symbiotic associations. In this study, we conducted phylogenetic analyses and characterization of T3SS genes, in addition to the synteny assignment of the gene clusters. Typically, the presence of more than one T3SS gene cluster within the same genome results from independent horizontal gene transfer events. Remarkably, despite the lack of canonical needle proteins, a putative SctF‐encoding CDS was found outside the main T3SS gene clusters, which is highly conserved in all analyzed genomes. These findings indicate a progressive coevolution of plant‐ and human‐associated systems, enabling adaptation to multiple hosts within the bacteria life cycle. Despite the widespread distribution of T3SS in *B. cenocepacia* isolates, strong evidence demonstrates that the T6SS is the main virulence factor in *B. cenocepacia* pathogenesis in human cells (Loutet and Valvano 2010; Monjarás Feria and Valvano 2020; Romero‐Gutiérrez et al. 2020; Spiewak et al. 2019). Therefore, these observations suggest a possible role of T3SS in the life cycle of *B. cenocepacia* by mediating its association with plants.

## Author Contributions

**Gabrielle Tomé Cordeiro:** conceptualization, writing – original draft, investigation, writing – review and editing. **Hadassa Loth de Oliveira:** investigation, writing – review and editing. **Danielly C. O. Mariano:** investigation, writing – review and editing. **Yasmin Salazar Torres:** investigation, writing – review and editing. **Graciela Maria Dias:** investigation, writing – review and editing, conceptualization. **Bianca C. Neves:** conceptualization, writing – original draft, funding acquisition, supervision.

## Ethics Statement

The authors have nothing to report.

## Conflicts of Interest

The authors declare no conflicts of interest.

## Supporting information


**Figure S1:** Geographical origin of the genomes. **Figure S2:** Variation in the genome size. **Figure S3:** Variation in CDS number. **Figure S4:** Variation in GC content (%). **Figure S5:** The putative *sctF* gene in different *B. cenocepacia* strains. **Table S1:** Strains of *Burkholderia cenocepacia* of environmental origin and their respective genomic information. **Table S2:** Strains of *Burkholderia cenocepacia* of clinical origin and their respective genomic information. **Table S3:** Distribution of type III secretion subtypes and genomic coordinates of the orphan *sctF*.
