# Supplementary material for: Phylogeny, Synteny, and Distribution of Type III Secretion Systems in Burkholderia cenocepacia: A Closer Look Into Host Span
Source: Microbiologyopen. 2025 Nov 2;14(6):e70101. doi: 10.1002/mbo3.70101 (PMC12580581; doi:10.1002/mbo3.70101)
Supplement: Supplementary file 1 — Figure S1: Geographical origin of the genomes. Figure S2: Variation in the genome size. Figure S3: Variation in CDS number. Figure S4: Variation in GC content (%). Figure S5: The putative sctF gene in different B. cenocepacia strains. Table S1: Strains of Burkholderia cenocepacia of environmental origin and their respective genomic information. Table S2: Strains of Burkholderia cenocepacia of clinical origin and their respective genomic information. Table S3: Distribution of type III secretion subtypes and genomic coordinates of the orphan sctF. [file MBO3-14-e70101-s001.docx]

**Phylogeny, Synteny, and Distribution of Type III Secretion Systems in *Burkholderia cenocepacia*: A Closer Look into Host Span**

Gabrielle Tomé Cordeiro^1^, Hadassa Loth de Oliveira^1^, Danielly C. O. Mariano^1^, Yasmin Salazar Torres^1^, Graciela Maria Dias^1†^ and Bianca C. Neves^1*^

***^1^****Instituto de Química, Universidade Federal do Rio de Janeiro, Brasil.*

****Corresponding author***

Bianca C. Neves

Instituto de Química, Universidade Federal do Rio de Janeiro (UFRJ)

Avenida Athos da Silveira Ramos, 149, A537

Rio de Janeiro, RJ, 21941-919, Brazil

Telephone: +55 21 3938-7355

e-mail: [bcneves@iq.ufrj.br](mailto:bcneves@iq.ufrj.br)

^†^**Present address**

*Instituto de Biofísica Carlos Chagas Filho, Universidade Federal do Rio de Janeiro, Brasil.*

| **Table S1: Strains of *Burkholderia cenocepacia* of environmental origin and their respective genomic information.** | | | | | | | | | | | |
| --- | --- | --- | --- | --- | --- | --- | --- | --- | --- | --- | --- |
| **Organism Name** | **Isolation Source** | **Strain** | **BioSample** | **BioProject** | **Assembly** | **Genome integrity** | **Genome Size(Mb)** | **GC%** | **Localization** | **Reference** | **Latitude, Longitude** |
| *Burkholderia cenocepacia* | water | MSMB384WGS | SAMN03449602 | PRJNA279182 | GCA_001718895.1 | Complete | 7.78060 | 67.2 | Australia, Northern Territory | 10.1371/journal.pntd.0003892 | -19.490000, 132.550000 |
| *Burkholderia cenocepacia* | soil | Bp8985 | SAMN09073238 | PRJNA451205 | GCA_003854725.1 | Contig | 7.39275 | 67.0 | Puerto Rico, Juncos | 10.1371/journal.pntd.0007727 | 18.227500, -65.920889 |
| *Burkholderia cenocepacia* | water | Bp9023 | SAMN09073273 | PRJNA451205 | GCA_003854055.1 | Contig | 7.33311 | 67.1 | Puerto Rico, Manati | 10.1371/journal.pntd.0007727 | 18.427694, -66.477694 |
| *Burkholderia cenocepacia* | soil | Bp8979 | SAMN09073235 | PRJNA451205 | GCA_003854115.1 | Contig | 7.55294 | 66.8 | Puerto Rico, Ceiba | 10.1371/journal.pntd.0007727 | 18.264194, -65.648306 |
| *Burkholderia cenocepacia* | soil | Bp8978 | SAMN09073234 | PRJNA451205 | GCA_003854125.1 | Contig | 7.55269 | 66.8 | Puerto Rico, Ceiba | 10.1371/journal.pntd.0007727 | 18.264194, -65.648306 |
| *Burkholderia cenocepacia* | soil | Bp9150 | SAMN09073390 | PRJNA451205 | GCA_003854485.1 | Contig | 7.51785 | 67.0 | Puerto Rico, Cabo Rojo | 10.1371/journal.pntd.0007727 | 18.086611, -67.145694 |
| *Burkholderia cenocepacia* | soil | Bp9134 | SAMN09073376 | PRJNA451205 | GCA_003854595.1 | Contig | 7.71317 | 67.2 | Puerto Rico, Maunabo | 10.1371/journal.pntd.0007727 | 18.007194, -65.899306 |
| *Burkholderia cenocepacia* | soil | Bp8970 | SAMN09073226 | PRJNA451205 | GCA_003854785.1 | Contig | 7.41251 | 67.1 | Puerto Rico, Ceiba | 10.1371/journal.pntd.0007727 | 18.264194, -65.648306 |
| *Burkholderia cenocepacia* | soil | Bp9127 | SAMN09073369 | PRJNA451205 | GCA_003854625.1 | Contig | 7.69277 | 66.6 | Puerto Rico, Cabo Rojo | 10.1371/journal.pntd.0007727 | 18.086611, -67.145694 |
| *Burkholderia cenocepacia* | soil | Bp9158 | SAMN09073398 | PRJNA451205 | GCA_003854475.1 | Contig | 7.78324 | 67.2 | Puerto Rico, Maunabo | 10.1371/journal.pntd.0007727 | 18.007194, -65.899306 |
| *Burkholderia cenocepacia* | soil | Bp9129 | SAMN09073371 | PRJNA451205 | GCA_003854585.1 | Contig | 7.69050 | 66.6 | Puerto Rico, Cabo Rojo | 10.1371/journal.pntd.0007727 | 18.086611, -67.145694 |
| *Burkholderia cenocepacia* | soil | Bp9145 | SAMN09073385 | PRJNA451205 | GCA_003854495.1 | Contig | 7.55000 | 67.0 | Puerto Rico, Cabo Rojo | 10.1371/journal.pntd.0007727 | 18.086611, -67.145694 |
| *Burkholderia cenocepacia* | soil | Bp8974 | SAMN09073230 | PRJNA451205 | GCA_003854775.1 | Contig | 7.76081 | 66.7 | Puerto Rico, Ceiba | 10.1371/journal.pntd.0007727 | 18.264194, -65.648306 |
| *Burkholderia cenocepacia* | soil | Bp9146 | SAMN09073386 | PRJNA451205 | GCA_003854505.1 | Contig | 7.46291 | 67.1 | Puerto Rico, Cabo Rojo | 10.1371/journal.pntd.0007727 | 18.086611, -67.145694 |
| *Burkholderia cenocepacia* | soil | Bp9139 | SAMN09073380 | PRJNA451205 | GCA_003854545.1 | Contig | 7.56367 | 67.4 | Puerto Rico, Arroyo | 10.1371/journal.pntd.0007727 | 17.965806, -66.061306 |
| *Burkholderia cenocepacia* | water | Bp9038 | SAMN09073284 | PRJNA451205 | GCA_003858255.1 | Contig | 7.63750 | 66.8 | Puerto Rico, Patillas | 10.1371/journal.pntd.0007727 | 18.006389, -66.015694 |
| *Burkholderia cenocepacia* | soil | Bp9117 | SAMN09073359 | PRJNA451205 | GCA_003854065.1 | Contig | 7.72518 | 66.8 | Puerto Rico, Rincon | 10.1371/journal.pntd.0007727 | 18.340194, -67.250000 |
| *Burkholderia cenocepacia* | water | Bp9037 | SAMN09073283 | PRJNA451205 | GCA_003858275.1 | Contig | 7.63914 | 66.8 | Puerto Rico, Patillas | 10.1371/journal.pntd.0007727 | 18.340194, -67.250000 |
| *Burkholderia cenocepacia* | soil | Bp9128 | SAMN09073370 | PRJNA451205 | GCA_003854605.1 | Contig | 7.53964 | 66.9 | Puerto Rico, Cabo Rojo | 10.1371/journal.pntd.0007727 | 18.086611, -67.145694 |
| *Burkholderia cenocepacia* | Rhizosphere, maize | MC0-3 | SAMN02598404 | PRJNA17929 | GCA_000019505.1 | Complete | 7.97139 | 66.6 | United States, Michigan | 10.1128/AEM.01941-07 | 44.182194, -84.506806 |
| *Burkholderia cenocepacia* | corn root | CR318 | SAMN05756084 | PRJNA342374 | GCA_002007585.1 | Complete | 7.66489 | 66.8 | Canada, London, Ontario | 10.1128/genomeA.00490-17 | 42.983611, -81.249694 |
| *Burkholderia cenocepacia* | onion field soil isolate | HI2424 | SAMN00113327 | PRJNA13918 | GCA_000203955.1 | Complete | 7.70284 | 66.8 | United States, New York | 10.1016/S0140-6736(02)08836-0 | 40.730611, -73.935194 |
| *Burkholderia cenocepacia* | cucumber root | APO9 | SAMN05178613 | PRJNA474611 | - | Complete | 8.05340 | - | Israel, Hazera | 10.1128/aem.02222 | 31.006000, 35.193472 |
| *Burkholderia cenocepacia* | agricultural soil | CEIB S5-2 | SAMN04252993 | PRJNA301637 | GCA_001541445.1 | Contig | 8.97605 | 65.7 | Mexico, Tepoztlan | 10.1128/genomeA.00220-16 | 31.006000, 35.193472 |

| **Table S2: Strains of *Burkholderia cenocepacia* of clinical origin and their respective genomic information.** | | | | | | | | | | | |
| --- | --- | --- | --- | --- | --- | --- | --- | --- | --- | --- | --- |
| **Organism Name** | **Isolation Source** | **Strain** | **BioSample** | **BioProject** | **Assembly** | **Genome integrity** | **Genome Size(Mb)** | **GC%** | **Localization** | **Reference** | **Latitude, Longitude** |
| *Burkholderia cenocepacia* | sputum | VC7848 | SAMN05001925 | PRJNA289138 | GCA_001999785.1 | Complete | 7.49946 | 66.9 | Canada, Vancouver | 10.1513/AnnalsATS.201408-395OC | 49.240000, -123.120000 |
| *Burkholderia cenocepacia* | cord blood | 895 | SAMN04574071 | PRJNA316047 | GCA_001606135.1 | Complete | 8.73148 | 66.7 | Malaysia, Kuala Lumpur | Unpublished | 3.181639, 101.692028 |
| *Burkholderia cenocepacia* | sputum | VC12802 | SAMN05001933 | PRJNA289138 | GCA_001999825.1 | Complete | 7.39491 | 67.0 | Canada, Vancouver | 10.1513/AnnalsATS.201408-395OC | 49.240000, -123.120000 |
| *Burkholderia cenocepacia* | sputum | J2315 | SAMEA1705928 | PRJNA339 | GCA_000009485.1 | Complete | 8.05578 | 66.9 | United Kingdom,Edinburgh | 10.1128/JB.01230-08 | 55.953306, -3.188306 |
| *Burkholderia cenocepacia* | nasal | 842 | SAMN04570263 | PRJNA315790 | GCA_001606115.1 | Complete | 8.14970 | 67.0 | Malaysia, Terengganu | Unpublished | 5.315944, 103.149222 |
| *Burkholderia cenocepacia* | sputum | H111 | SAMEA3138403 | PRJNA69823 | GCA_000236215.4 | Complete | 7.71489 | 67.3 | Germany, Hannover | 10.1111/j.1574-6968.2000.tb09026.x | 52.373889, 9.735611 |
| *Burkholderia cenocepacia* | sputum | ST32 | SAMN03323790 | PRJNA274219 | GCA_001484665.1 | Complete | 8.09039 | 67.0 | Czech Republic | 10.1099/jmm.0.46025-0 | 50.070000, 14.340000 |
| *Burkholderia cenocepacia* | sputum | VC12308 | SAMN05001912 | PRJNA289138 | GCA_001999885.1 | Complete | 7.63450 | 67.1 | Canada, Vancouver | 10.1513/AnnalsATS.201408-395OC | 49.240000, -123.120000 |
| *Burkholderia cenocepacia* | sputum | PC184 Mulks | SAMN06842015 | PRJNA384579 | GCA_003076415.1 | Complete | 7.06705 | 66.8 | United States, Ohio | Unpublished | 41.490000, -81.690000 |
| *Burkholderia cenocepacia* | blood | AU 1054 | SAMN02598326 | PRJNA13919 | GCA_000014085.1 | Complete | 7.27912 | 66.9 | United States | 10.1186/s12866-017-0986-6 | 42.338639, -71.103167 |
| *Burkholderia cenocepacia* | sputum | VC2307 | SAMN05001798 | PRJNA289138 | GCA_001999805.1 | Chromosome | 7.91588 | 67.0 | Canada, Vancouver | 10.1513/AnnalsATS.201408-395OC | 49.240000, -123.120000 |
| *Burkholderia cenocepacia* | sputum | VC1254 | SAMN05001969 | PRJNA289138 | GCA_001999925.1 | Chromosome | 8.17737 | 67.0 | Canada, Vancouver | 10.1513/AnnalsATS.201408-395OC | 49.240000, -123.120000 |
| *Burkholderia cenocepacia* | sputum | GIMC4560:Bcn122 | SAMN06611151 | PRJNA379546 | GCA_002083015.1 | Chromosome | 8.04369 | 67.1 | Russia, Moscow | 10.1186/s12864-018-4472-9 | 55.768111, 37.466556 |
| *Burkholderia cenocepacia* | clinical isolate | FDAARGOS 518 | SAMN10163212 | PRJNA231221 | GCA_003940705.1 | Contig | 7.49941 | 67.4 | United States, Washington | 10.1038/s41467-019-11306-6 | 38.980000, -77.110000 |
| *Burkholderia cenocepacia* | sputum | VC7604 | SAMN05001827 | PRJNA289138 | GCA_001984275.1 | Contig | 7.09484 | 66.9 | Canada, Vancouver | 10.1513/AnnalsATS.201408-395OC | 49.240000, -123.120000 |
| *Burkholderia cenocepacia* | sputum | FDAARGOS 187 | SAMN05004742 | PRJNA231221 | GCA_002891255.1 | Contig | 7.46533 | 67.4 | United States, Washington | 10.1038/s41467-019-11306-6 | 38.980000, -77.110000 |
| *Burkholderia cenocepacia* | sputum | VC9789 | SAMN05001819 | PRJNA289138 | GCA_001984315.1 | Contig | 7.80352 | 66.9 | Canada, Vancouver | 10.1513/AnnalsATS.201408-395OC | 49.240000, -123.120000 |
| *Burkholderia cenocepacia* | sputum | VC4558 | SAMN05001820 | PRJNA289138 | GCA_001984325.1 | Contig | 8.22889 | 67.0 | Canada, Vancouver | 10.1513/AnnalsATS.201408-395OC | 49.240000, -123.120000 |
| *Burkholderia cenocepacia* | sputum | FDAARGOS 189 | SAMN05004744 | PRJNA231221 | GCA_002891135.1 | Contig | 8.09455 | 66.9 | United States, Washington | 10.1038/s41467-019-11306-6 | 38.980000, -77.110000 |
| *Burkholderia cenocepacia* | sputum | VC5486 | SAMN05001975 | PRJNA289138 | GCA_001984285.1 | Contig | 7.73614 | 66.9 | Canada, Vancouver | 10.1513/AnnalsATS.201408-395OC | 49.240000, -123.120000 |
| *Burkholderia cenocepacia* | sputum | VC3917 | SAMN05001990 | PRJNA289138 | GCA_001984355.1 | Contig | 7.34467 | 66.8 | Canada, Vancouver | 10.1513/AnnalsATS.201408-395OC | 49.240000, -123.120000 |
| *Burkholderia cenocepacia* | sputum | FDAARGOS 82 | SAMN02700076 | PRJNA231221 | GCA_000783555.2 | Contig | 7.31971 | 66.9 | United States, Washington | 10.1038/s41467-019-11306-6 | 38.980000, -77.110000 |
| *Burkholderia cenocepacia* | sputum | VC1255 | SAMN05001970 | PRJNA289138 | GCA_001984375.1 | Contig | 8.37363 | 66.9 | Canada, Vancouver | 10.1513/AnnalsATS.201408-395OC | 49.240000, -123.120000 |
| *Burkholderia cenocepacia* | sputum | K56-2Valvano | SAMN00255233 | PRJNA62783 | GCA_000333155.2 | Contig | 7.75026 | 67.0 | Canada, Toronto | 10.1128/iai.66.2.874-877.1998 | 43.651000, -79.347000 |

| **Table S3: Distribution of type III secretion subtypes and genomic coordinates of the orphan *sctF*.** | | | | | | | | | | | |
| --- | --- | --- | --- | --- | --- | --- | --- | --- | --- | --- | --- |
| ***Burkholderia cenocepacia* strain** | **T3SS subtype** | | | | | | | | | **Genomic coordinates of a putative *sctF* (needle protein gene) E-value 10e-5** | **Putative SctF aminoacid sequence** |
|  | **1** | **2** | **3** | **4** | **5** | **6** | **7** | **8** | **9** |  |  |
| Bc_842 |  | **X** |  |  |  |  |  |  |  | 2436194..2445289 | MSEQIKHISDASFEQDVVKSDKPVLVDFWAEWCGPCKMIAPILDEVAKDYGDKLQIAKINVDDNQATPAKFGVRGIPTLILFKNGAAAAQKVGALSKSQLTAFLDSHL |
| Bc_895 |  | **X** |  |  |  |  |  |  |  | 3673484..3683362 |  |
| Bc_APO9 |  |  |  |  |  |  | **X** |  |  | 5346914..5357038 |  |
| Bc_AU_1054 |  | **X** |  |  |  |  |  |  |  | 874316..884497 |  |
| Bc_Bp8970 |  | **X** |  |  |  |  |  |  |  | 40171..50822 |  |
| Bc_Bp8974 |  | **X** |  |  |  |  |  | **X** |  | 38340..48504 |  |
| Bc_Bp8978 |  | **X** |  |  |  |  |  |  |  | 40578..52166 |  |
| Bc_Bp8979 |  | **X** |  |  |  |  |  |  |  | 40578..52166 |  |
| Bc_Bp8985 |  | **X** |  |  |  |  |  |  |  | 40151..50812 |  |
| Bc_Bp9023 |  | **X** |  |  |  |  |  |  |  | 40158..50804 |  |
| Bc_Bp9037 |  | **X** |  |  |  |  |  | **X** |  | 167025..177197 |  |
| Bc_Bp9038 |  | **X** |  |  |  |  |  | **X** |  | 167897..178176 |  |
| Bc_Bp9117 |  | **X** |  |  |  |  |  | **X** |  | 39626..48920 |  |
| Bc_Bp9127 |  | **X** |  |  |  |  |  |  |  | 257960..267846 |  |
| Bc_Bp9128 |  | **X** |  |  |  |  |  | **X** |  | 27112..37284 |  |
| Bc_Bp9129 |  | **X** |  |  |  |  |  |  |  | 257475..267361 |  |
| Bc_Bp9134 |  | **X** |  |  |  |  |  |  |  | 45511..55286 |  |
| Bc_Bp9139 |  | **X** |  |  |  |  |  |  |  | 38311..48435 |  |
| Bc_Bp9145 |  | **X** |  |  |  |  |  | **X** |  | 247214..256754 |  |
| Bc_Bp9146 |  | **X** |  |  |  |  |  | **X** |  | 245705..255604 |  |
| Bc_Bp9150 |  | **X** |  |  |  |  |  |  |  | 40578..52166 |  |
| Bc_Bp9158 |  | **X** |  |  |  |  |  |  |  | 46008..56324 |  |
| Bc_CEIB_S5_2 |  | **X** |  |  |  |  |  | **X** |  | 163475..173116 |  |
| Bc_CR318 |  | **X** |  |  |  |  |  |  |  | 2096171..2105883 |  |
| Bc_FDAARGOS_187 |  | **X** |  |  |  |  |  |  |  | 1528162..1538286 |  |
| Bc_FDAARGOS_189 | **X** |  |  |  |  |  |  |  |  | 759458..769335 |  |
| Bc_FDAARGOS_518 |  | **X** |  |  |  |  |  |  |  | 2896413..2906023 |  |
| Bc_FDAARGOS_82 |  | **X** |  |  |  |  |  |  |  | 1640772..1652345 |  |
| Bc_GIMC4560_Bcn122 |  |  |  |  | **X** |  |  |  |  | 1782172..1792337 |  |
| Bc_H111 |  |  |  |  |  | **X** |  |  |  | 1974610..1984775 |  |
| Bc_HI2424 |  | **X** |  |  |  |  |  |  |  | 2036357..2046075 |  |
| Bc_J2315 | **X** |  |  |  |  |  |  |  |  | 2099476..2109236 |  |
| Bc_K56_2Valvano |  | **X** |  |  |  |  |  |  |  | 24151..33246 |  |
| Bc_MC0_3 |  | **X** |  |  |  |  |  |  | **X** | 2064150..2073860 |  |
| Bc_MSMB384WGS |  | **X** |  |  |  |  |  |  |  | 2084478..2094236 |  |
| Bc_PC184_Mulks |  |  | **X** |  |  |  |  |  |  | 3118592..3128008 |  |
| Bc_ST32 |  |  |  | **X** |  |  |  |  |  | 1194440..1206013 |  |
| Bc_VC12308 |  | **X** |  |  |  |  |  |  |  | 1979613..1989388 |  |
| Bc_VC1254 |  |  |  | **X** |  |  |  |  |  | 2181286..2191061 |  |
| Bc_VC1255 |  |  |  | **X** |  |  |  |  |  | 653635..663338 |  |
| Bc_VC12802 |  | **X** |  |  |  |  |  |  |  | 942401..952539 |  |
| Bc_VC2307 |  |  |  | **X** |  |  |  |  |  | 3197681..3207438 |  |
| Bc_VC3917 |  |  |  | **X** |  |  |  |  |  | 2025742..2035801 |  |
| Bc_VC4558 |  | **X** |  |  |  |  |  |  |  | 2936334..2945785 |  |
| Bc_VC5486 |  |  |  | **X** |  |  |  |  |  | 1070027..1080483 |  |
| Bc_VC7604 |  | **X** |  |  |  |  |  |  |  | 462947..472824 |  |
| Bc_VC7848 |  | **X** |  |  |  |  |  |  |  | 2601658..2611532 |  |
| Bc_VC9789 |  |  |  |  |  |  |  |  |  | 2217090..2226847 |  |


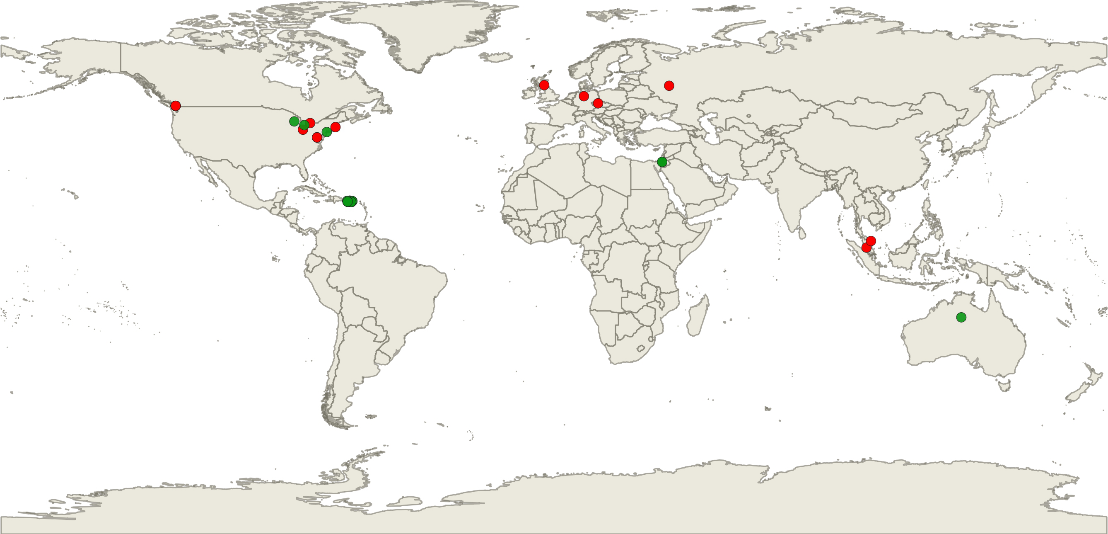


**Figure S1. Geographical origin of the genomes.** Red dots indicate clinical origin, and green dots represent environmental origin.


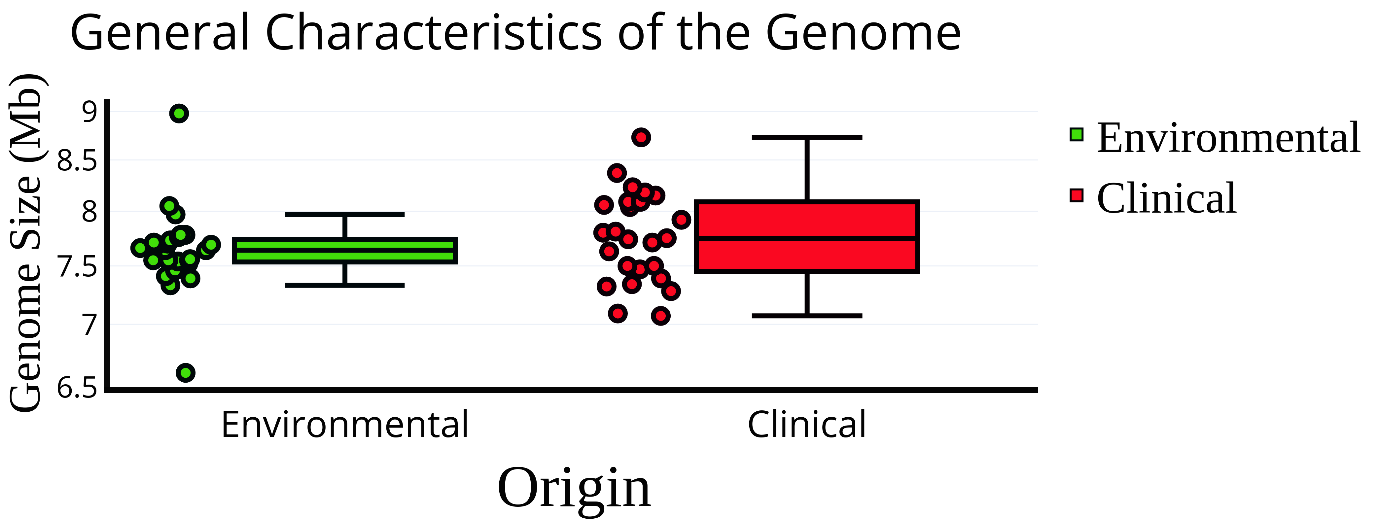


**Figure S2. Variation in the genome size.** The average sizes of environmental and clinical genomes are not significantly different (p < 3.55E-01).


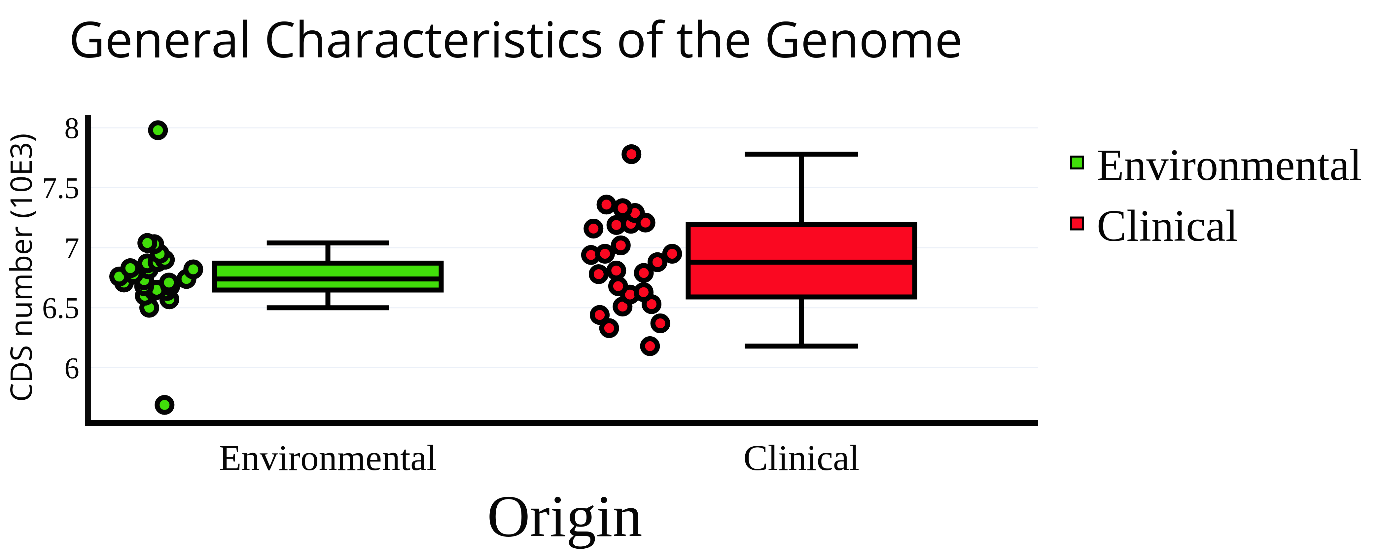


**Figure S3.** **Variation in CDS number.** The average CDS numbers in environmental and clinical genomes are not significantly different (p < 1.68E-01).


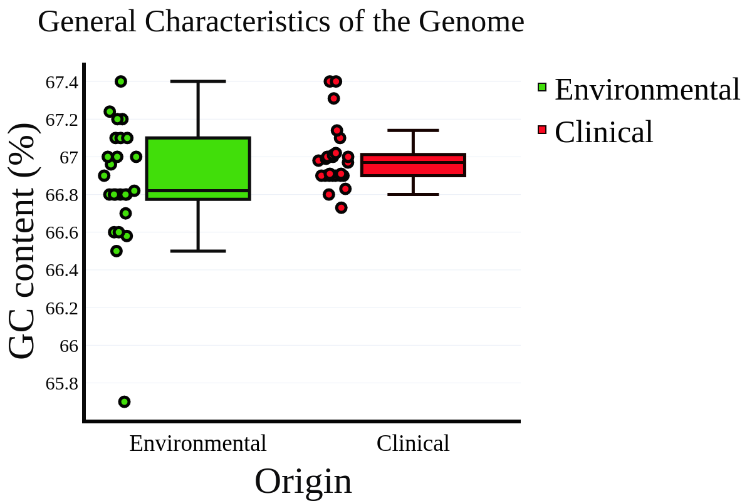


**Figure S4.** **Variation in GC content (%).** The average GC content (%) in environmental and clinical genomes are not significantly different (p < 1.14E-01).


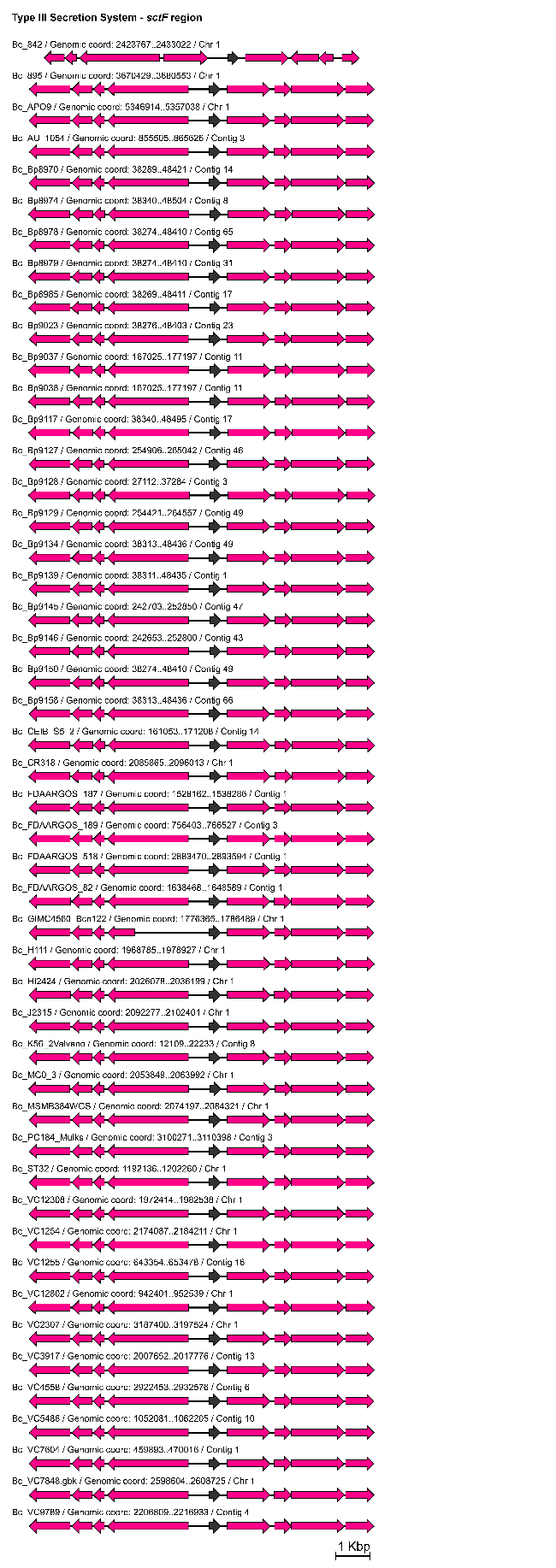


**Figure S5. The putative *sctF* gene in different *B. cenocepacia* strains.** Analyses revealed a conserved CDS located outside the main T3SS clusters, encoding a needle-like protein (*sctF*, in black). The amino acid sequence is identical across all strains analyzed. Neighboring genes are shown in pink.
